# Fibula Free Flap Elevation without Tourniquet: Are Harmonic Scalpel Shears Useful?

**DOI:** 10.1097/GOX.0000000000002409

**Published:** 2019-09-10

**Authors:** Marta Starnoni, Giorgio De Santis, Massimo Pinelli

**Affiliations:** From the Division of Plastic Surgery, University of Modena and Reggio Emilia, Largo Pozzo 71, 41124 Modena, Italy.

Harmonic Scalpel (HS, Ethicon Endo-Surgery, Cincinnati, Ohio) shears represent an alternative to traditional techniques of free flap elevation. This multifunctional device presents a few advantages when compared with traditional electrocoagulation, including simultaneous tissue dissection and hemostasis, no eschar formation over the blade, minimal thermal damage, no smoke formation, and possibility of use in patients with pacemaker (1).

As far as head and neck reconstruction is concerned, the literature is uncertain, with some studies showing reduced operative time with HS (2,4) and others the opposite (3,5). Nevertheless it is considered to be a reliable, safe, and alternative method of free flap harvesting in cranio-facial reconstructive surgery (3,5).

**Fig.1.**
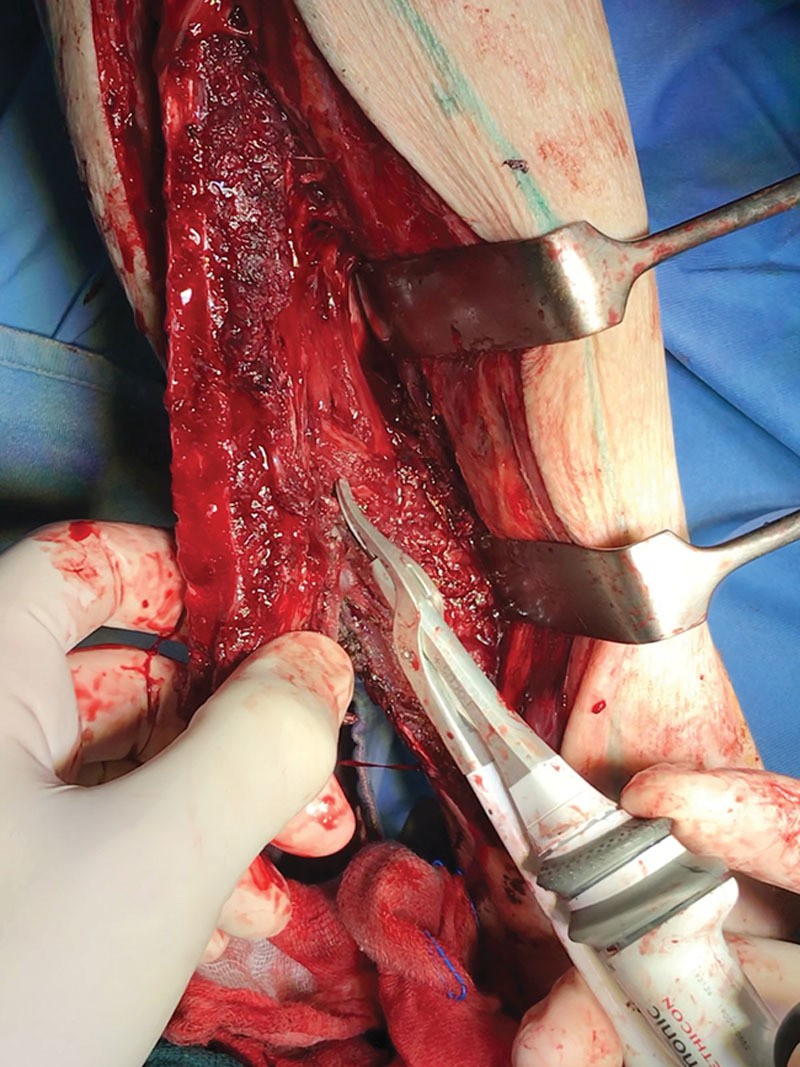
Elevation of the fibula free flap without tourniquet using Harmonic Scalpel shears.

Fibula free flap elevation using HS has been reported under tourniquet only (2–5). We have experienced this surgical device for the elevation of the fibula free flap without tourniquet (Fig. 1). In fact, for what concerns the use of tourniquet, our experience shows that it is not required in fibula free flap harvesting because there are not specific advantages. Moreover, tourniquet is known to be a risky procedure because it can induce micro-thromboses, muscle edema, and nerve-related injury due to local compression. In addition, no pulse of the skin paddle perforators is visible and selection of the most suitable vessel can be difficult, especially for microsurgical trainees. In the perfused leg, anatomic structures are easily identified and bleeding is controlled permanently.

We compared 2 groups of 18 patients: group A (elevation of the free fibula flap with HS) and group B (elevation of the free fibula flap with electrocautery). Both groups were operated on by a senior and a trainee and without tourniquet.

The most important differences between the 2 groups were about the length of time until drain removal (second postoperative day in group A and fourth postoperative day in group B) and the flap elevation operative time (57 minutes in group A and 83 minutes in group B). Despite these results, in our opinion these differences are not clinically relevant. Flap raising is always performed simultaneously with head and neck resection, which usually is more time-consuming. This means that a reduction of the operation time, mandatory with the use of a tourniquet, is not so necessary if the operation is performed in a perfused leg. Furthermore, the day of the drain removal (second or fourth) is not a critical point because daily patient ambulation is limited by general conditions and the presence/absence of drain is not an issue.

Nevertheless, in our opinion the Harmonic Scalpel has the advantage of an immediate and meticulous hemostasis, which is mandatory when raising the flap without a tourniquet. Furthermore, and less importantly, the use of Harmonic Scalpel improves self- confidence in our microsurgical trainees during the fibula flap elevation without tourniquet, because accurate haemostasis allows for better visualisation of anatomic structures. The use of the monopolar cautery causes muscle contraction with the risk of unwanted vascular injury, and the electric energy can potentially injure the vascular bundle.

Despite limited evidence, in our opinion this device could be useful for fibula free flap elevation without tourniquet, especially if the surgeon is a microsurgical trainee.
